# First serological and molecular investigation of hepatitis E virus infection in dromedary camels in Algeria

**DOI:** 10.3389/fvets.2023.1272250

**Published:** 2023-09-19

**Authors:** Amir Agabou, Mohamed Hocine Benaissa, Ilyes Bouasla, Luca De Sabato, Sana Hireche, Giovanni Ianiro, Marina Monini, Ilaria Di Bartolo

**Affiliations:** ^1^PADESCA Research Laboratory, Institute of Veterinary Sciences, University Frères Mentouri Constantine 1, Constantine, Algeria; ^2^Scientific and Technical Research Centre for Arid Areas (CRSTRA), Touggourt, Algeria; ^3^Department of Food Safety, Nutrition and Veterinary Public Health, Istituto Superiore di Sanità, Rome, Italy

**Keywords:** hepatitis E virus, ELISA, seroprevalence, risk factors, dromedary camels, Algeria

## Abstract

Hepatitis E is an acute self-limited or fulminant infection in humans, caused by the hepatitis E virus (HEV). This member of the *Hepeviridae* family has been identified in a wide range of domestic and wild animals all over the world, with a possible transmission to humans through fecal oral route, direct contact and ingestion of contaminated meat products, making it one of the global zoonotic and public health major concerns. Since there is no monitoring program and a lack of data on HEV in animals in Algeria, the current preliminary survey has been undertaken to elucidate the exposure to the virus in camels at abattoirs of six southern provinces of Algeria. Two-hundred and eight sera/plasma were collected and analyzed (by double antigen sandwich ELISA) for the presence of total anti-HEV antibodies, among which 35.1% were positive, but no HEV RNA could be isolated from them (by two pan-HEV nested RT-PCR and broad range real-time reverse transcription RT-PCR). The univariate analysis showed significant associations (*p* < 0.05) between HEV seroprevalence and province of origin, age, and sex of camels, whereas the multivariable logistic regression analysis revealed a negative impact of camels’ age on it. The obtained results confirm that HEV infection is widespread established in the camelid population of Algeria.

## 1. Introduction

Hepatitis E virus (HEV) is an icosahedral and single-stranded positive sense polarity RNA virus. The virions are shed naked in feces and bile, and they circulate quasi-enveloped (cloaked in a lipid envelope) in blood (1).

Hepatitis E virus belongs to the *Hepeviridae* family and to the genus *Orthohepevirus*. This last is divided into four species, named *Orthohepevirus A* to *D*. *Orthohepevirus A* encompasses eight distinct genotypes (HEV-1 to HEV-8) and several sub-genotypes (2). Hepatitis E virus-1 and HEV-2 infect only humans, HEV-3 and HEV-4 humans and several mammal species (mainly suidae and less frequently red deer and roe deer), HEV-5 and HEV-6 wild boars, HEV-7 dromedary camels and humans, and HEV-8 Bactrian camels and long-tailed macaques (3–6).

Hepatitis E virus is one of the five known hepatotropic viruses in humans (7). According to Li et al. (8), it is responsible of an acute self-limiting hepatitis, and it infects about 939 million individuals all around the world. Every year, 20 million new HEV infections, with 3.3 million acute clinical cases and over than 44.000 related deaths are reported worldwide (9). Hepatitis E virus-1 and HEV-2 are epidemic and endemic in developing countries in Asia and Africa, while HEV-3 and HEV-4 are sporadic in developed countries in Europe, North America and Japan (10–13), causing a variety of hepatic and extrahepatic manifestations (14). Hepatitis E virus-3, 4 and 7 infections are zoonotic (15) transmitted between animals and humans by consumption of raw or undercooked meat (HEV3 and 4) and milk (HEV-7) or contact with infected animals (16).

All over the world, the seroprevalence of HEV infection in dromedary camels range from 8.3 to 62.9% (17, 18). In Algeria, there are about 416.500 camels (*Camelus dromedarius*) raised following three breeding systems (sedentary, nomadic, and transhumant) in 9 Steppic and 8 Saharan provinces (19), where they occupy a crucial place in sustaining the livelihood of local and nomadic populations by participating to their food security, facilitating their displacements, and providing household incomes. In addition, they play other cultural and social functions such as utilization in some religious feasts and traditional celebrations, racing, gaming, exhibitions and some recreational activities. This close contact with people will increase the likelihood of transmitting many zoonotic infectious agents (including HEV) through all possible routes.

Review of the literature revealed scarce information regarding HEV infection in camels all over the world and none in Algeria. Thus, we have undertaken this preliminary study to elucidate the seroprevalence of this infection and some of its associated risk factors in the camelid population of this country.

## 2. Materials and methods

### 2.1. Epidemiological data and samples collection

**Figure 1:**
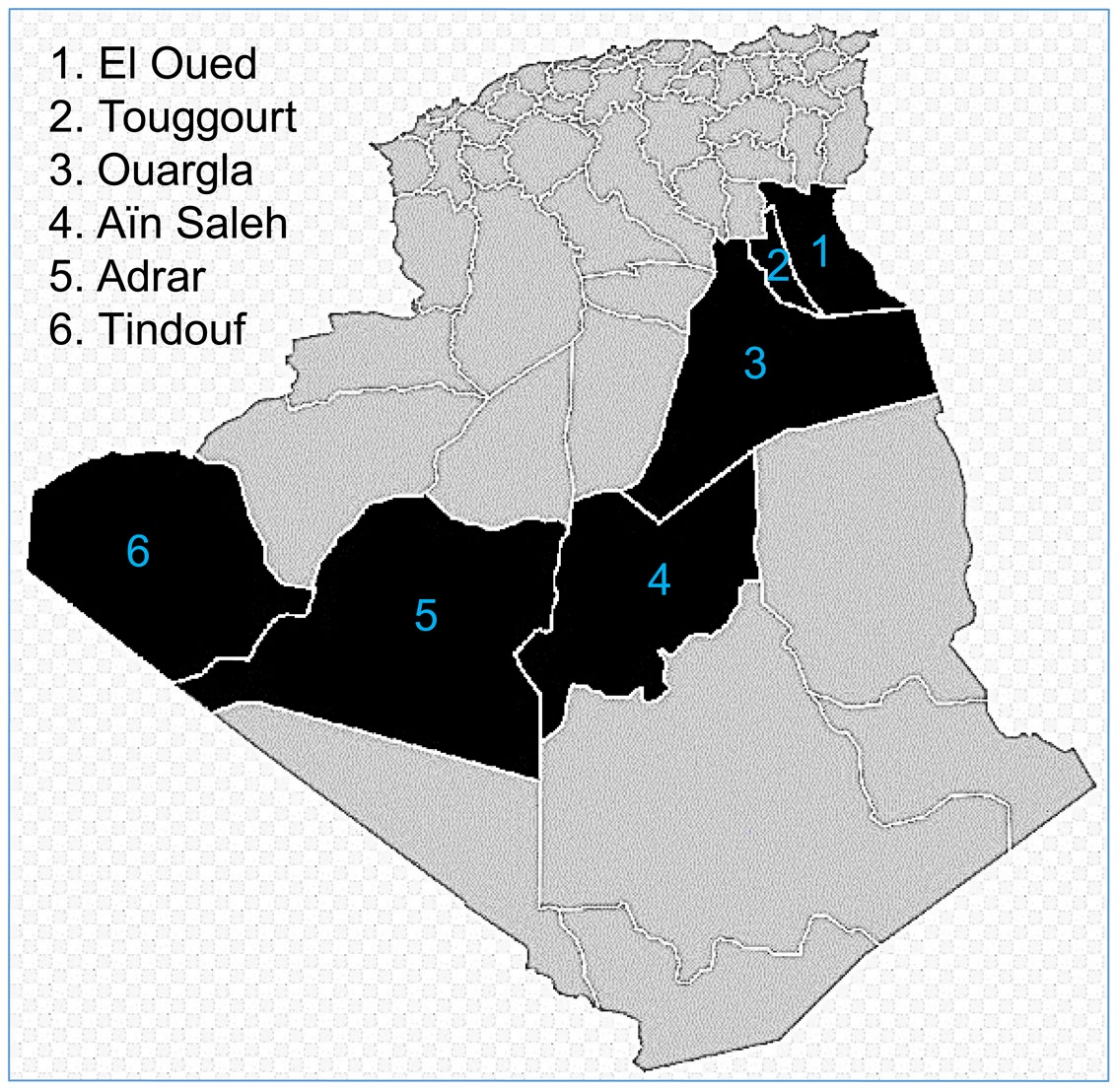
Sampling provinces from Algerian Sahara.

From June to December 2019 and January to March 2022, 208 apparently healthy camels (*Camelus dromedarius*) were sampled at slaughterhouses of six southern provinces of Algeria (Figure 1): Adrar (*n* = 30), Aïn Saleh (*n* = 15), El Oued (*n* = 102), Ouargla (*n* = 22), Tindouf (*n* = 30), and Touggourt (*n* = 9).

On arrival at the abattoirs, blood samples were taken aseptically from the jugular vein, either in EDTA tubes or in tubes with clot accelerator and gel serum separator. All procedures performed in this study were in accordance with the ethical standards of the Scientific and Ethics Committee for Animal Experimentation of the Institute of veterinary sciences, University Frères Mentouri of Constantine 1 (Algeria).

Plasma/sera were then separated by centrifugation at 3,000 × g for 5 min and stored at −20°C until assayed.

Since animals to be slaughtered are brought by dealers or butchers and not by herds’ owners, only few epidemiological data (breed, age, and sex in addition to province of origin) could be successfully collected. Sampled animals were 83 males and 125 females belonging to the two most dominant breeds in Algeria: Sahraoui (*n* = 91) and Targui (*n* = 117). Their ages ranged between 1 and 28 years, and they were grouped into 5 age strata (in years): [1–6] (*n* = 69), [7–12] (*n* = 62), [13–18] (*n* = 47), [19–24] (*n* = 24) and [25–28] (*n* = 6).

### 2.2. Laboratory analysis

The plasma/serum samples were tested by a double antigen sandwich ELISA kit (HEV ELISA 4.0v. MP Diagnostics-Biomedicals, Eschwege, Germany), developed exclusively for veterinary use, and validated to identify total antibodies (IgM, IgG, and IgA), directed against HEV recombinant protein ET2.1 derived from the ORF2 capsid protein (20–22), in all animal species’ plasma or sera. Both sensitivity and specificity of this test are established at 99.2%.

Optical densities (ODs) were measured at 450 nm using an Infinite F50 absorbance microplate reader (Tecan Group Ltd., Zurich, Switzerland). As specified by the manufacturer’s validation criteria, a sample is considered positive when its OD is higher than the threshold defined as the mean OD for negative controls + 0.3.

### 2.3. Nucleic acid detection for Hepatitis E virus

Total RNAs from positive sera were extracted using QIAmp Viral RNA Mini Kit (Qiagen, Monza, Italy) on a QIAcube Connect automated extraction platform as per the manufacturer’s instructions, then mixed in pools of six samples, prior of being analyzed by two pan-HEV nested RT-PCR as reported by Boxman et al. (23) and Drexler et al. (24). PCR products were separated on a 1.0% agarose gel, stained with GelRed Nucleic Acid Gel Stain (Biotium Inc., Hayward, CA, United States) and visualized under UV light. Next, samples were also subjected to the broad range real-time reverse transcription RT-PCR described by Jothikumar et al. (25) for HEV-1-4 detection.

### 2.4. Statistical analysis

Data were processed and analyzed using SPSS 25.0 software (SPSS Inc., Chicago, IL, United States, 2017). True prevalence was calculated using the Rogan-Gladen estimator (26).

The associations of various risk factors to the camels’ HEV serological status were first screened by univariate analysis at *p* < 0.05 using contingency tables and odds ratios (OR) with lower and upper limits of the 95.0% confidence interval (CI). Potential factors showing moderate associations at *p* < 0.25 via the initial Pearson’s Chi-square test analysis were considered eligible for inclusion in the further multivariable logistic regression analysis. The analysis begins with a full or saturated model that was rerun by eliminating variables from it in an iterative process. The fit of the final model was evaluated by the Hosmer and Lemeshow goodness-of-fit test (27).

## 3. Results

An overall apparent anti-HEV seroprevalence of 35.1% was found, corresponding to a true prevalence of 34.8%. As shown in Table 1, the univariate analysis revealed that the factors province of origin, sex and age strata have a significant association with HEV seropositivity in camels (*p* = 0.034, *p* = 0.042, and *p* = 0.026, respectively). In fact, 50.7% of positive animals were females and 49.3% were males; however, 43.3% of sampled males were positive against 29.6% of sampled females. The infection was significantly more frequent in camels from Adrar province (53.3%) followed by Ouargla (50.0%) and Ain Saleh (43.3%) than the other provinces. As well, camels of the Targui breed yielded more seropositivity (38.5%) as compared to those of the Sahraoui one (30.8%), but not significantly. Concerning animals’ age, young camels of 1–6 years old were significantly the most seropositive (47.8%).

**Table 1:** Univariate analysis of the investigated risk factors associated with hepatitis E virus seropositivity among the sampled camels.

Variable	Category	Presence of antibodies		OR (95.0% CI)	*P* value
		Negative *n* (%)	Positive *n* (%)		
Province of origin	Adrar	14 (46.7)	16 (53.3)	12.06 (ND)	0.034*
	Aïn Saleh	8 (53.3)	7 (46.7)		
	El Oued	72 (70.6)	30 (29.4)		
	Ouargla	11 (50.0)	11 (50.0)		
	Tindouf	22 (73.3)	8 (26.7)		
	Touggourt	8 (88.9)	1 (11.1)		
Sex	Male	47 (56.6)	36 (43.4)	4.154 (0.307–0.980)	0.042*
	Female	88 (70.4)	37 (29.6)		
Breed	Sahraoui	63 (69.2)	28 (30.8)	1.330 (0.787–2.513)	0.249*
	Targui	72 (61.5)	45 (38.5)		
Age strata (years)	1–6	36 (52.2)	33 (47.8)	11.080 (ND)	0.026*
	7-12	40 (64.5)	22 (35.5)		
	13–18	38 (80.9)	9 (19.1)		
	19–24	16 (66.7)	8 (33.3)		
	25–28	5 (83.3)	1 (16.7)		

*Significant univariately at *p* < 0.25 and offered to the multivariable logistic regression model. ND: Not determined.

When subjected to the logistic regression analysis, only age strata still having a negative impact on the seropositivity (Table 2).

**Table 2:** Multivariable logistic regression analysis of factors associated with HEV seropositivity.

Variables	B^a^	SE^b^	OR^c^ (95.0% CI^d^)	*P* value
Constant	0.687	0.440	–	0.119
Age strata	−0.312	0.145	0.732 (0.551–0.972)	0.031

Model chi-square with *df* of 7. Model-2 log likelihood 259.139. Chi square goodness to fit = 9.932; *p* = 0.192. a. Logistic regression coefficient, b. Standard error, c. Odds ratio, and d. Confidence interval.

Finally, all the attempts to detect viral RNA in pools of HEV-positive sera were unsuccessful.

## 4. Discussion

To the best of our knowledge, this is the first survey to assess HEV seroprevalence in one-humped camels and its associated risk factors in Algeria.

The study revealed that 35.1% of camels tested positive for HEV antibodies. This value is higher than the 22.4 and 23.1% seroprevalences detected in Ethiopia and Saudi Arabia, respectively (28, 29). It is almost of the same level as the rates reported in this animal species in Nigeria (30.7%) (30), Kenya (31.4%), United Arab Emirates (37.1%), Somalia (40.0%), and Sudan (42.9%) (18). However, it is lower than the ones found in Israel (68.6%) by Bassal et al. (31) and in Egypt (62.9%) by Rasche et al. (18). Until now, no data are available for this infection in camels of the surrounding countries, apart from the work of Ouoba et al. (17) in the Sahelian zone of West Africa, where the seroprevalence was very low (8.3%) when compared to ours. This region includes, besides Burkina Faso, two other countries (Mali and Niger) that border Algeria from the southwest and the southeast. Differences in HEV prevalence between countries may result from many associations of factors that commonly affect several infectious diseases in camels (32, 33) and by factors associated to HEV epidemiology (34, 35). These latter factors are related to geographic and climatic disparities, camels’ husbandry and breeding systems, herds’ health management strategies (particularly biosecurity measures and sanitation), animals’ traits (sex, age, and health status), feed, and grazing management. In addition to other factors, specifically related to HEV, that should be investigated and identified in further studies. For instance, farming practices, such as extensive farming, absence of sanitary ford and quarantine period, and contact with domestic species have been identified as risk factors for HEV occurrence in pigs (34, 35).

In perfect accordance with the outcomes of our work, the impact of camels’ sex on HEV seropositivity has been well established in favor of males by El-Kafrawy et al. (28) and Sarani et al. (36). Conversely, in the studies by Li et al. (29) and Ouoba et al. (17), no statistically significant relationship between HEV seropositivity in camels and their sex was notified. The role of sexual dimorphism in the epidemiology of HEV in dromedary camels is not well established. Some physiological and molecular mechanisms could make males more predisposed to the infection than females. This risk factor deserves to be investigated further.

The fact that camels imported from Sudan and Djibouti were slightly less seropositive than Saudi Arabia’s domestic camels (28) and those imported from Afghanistan and Pakistan were more infected than Iranian local camels (36), implies a geographic (country) influence on the HEV distribution. This effect could not be ascertained by Ouoba et al. (17), who observed a random distribution of HEV-positive camels among the three countries of their study. We confirmed in our work an impact of province (county) of origin on the spread of this infection; however, similar surveys are unavailable to examine it, except the paper published by Sarani et al. (36) in Iran. Remarkably, geographic differences in HEV seroprevalence rates of cattle within different localities or provinces of the same country have already been demonstrated in Egypt and China (37, 38).

It is noteworthy that unrestrained camels’ displacements with nomadic families may contribute to the spread of this infection. These movements are very frequent across the Algerian borders (of about 6.500 km long), especially in the Southern provinces. Camels and other animals’ herds/flocks move through different countries for grazing lands and watering points where they mix and get contact with other domestic and wild animals that may contribute to the dissemination of this virus. Furthermore, within these regions, animals are sold in traditional marketplaces, where no biosecurity measures are applied, and several livestock coming from different herds and regions, congregate together with very close contact allowing contagion and spread of several infectious agents. As well, the possibility that humans may transfer the infection to camels cannot be excluded in these regions, since both animals and humans share the same HEV genotype (HEV-7) (5). Because of the non-enveloped nature of HEV outside its host, it resists and stays potentially infectious in water, soil, and grass for long periods (39).

El-Kafrawy et al. (28) and Ouoba et al. (17) could not find any effect of age on HEV seroprevalence in their sampled camels, in contrast to Bassal et al. (31) who recoded a prevalence of 88.9% in camels aged 10 years and more, 56.0% in those of 7 years old and 67.4% in younger ones (3 years old). In addition, Li et al. (29) and Sarani et al. (36) reported young camels of 2 and 3 years old and those less than 2 years old as the most frequently positive (40.0, 36.4, and 100%, respectively). These last results corroborate well with ours, as we have recorded a significant high prevalence among camels aged of 1–6 years old (47.8%). The high prevalence within this age stratum could be related to the sex of animals (males) and their origin. Indeed, this age section (1–6 years old) is largely composed of males, which are significantly more seropositive than females. Furthermore, it also contains the largest proportion of males of the whole study as compared to the other age strata. As well, it is composed of animals belonging to provinces where the prevalence rates are significantly higher as compared to the others (Adrar, Ouargla vs. El Oued, Tindouf, and Touggourt). According to Yugo et al. (40), absence of association between age and HEV seropositivity in calves and adult cows may be due to transient seroconversion. Corman et al. (41) reported that camels get HEV-7 infection in the first 6 months of life and clear the virus after 2 months approximately, making young animals more seropositive than elder ones, just like in our study.

As far as it could be found out, no information is accessible on the impact of camels’ breeds on the anti-HEV prevalence. According to Al Ramadan et al. (42), there is no evidence of particular resistance or susceptibility of certain camel breeds to some infections (may be hepatitis E is one of them).

Positive slaughtered animals yield contaminated meat and offal that can transfer the virus through direct contact to slaughterhouse workers, veterinarians, butchers, and consumers (43). Slot et al. (44), and Obaidat and Roess (45) described meat consumption as a major risk for HEV infection. In order to neutralize this risk and completely inactivate HEV, Barnaud et al. (46) recommend heating food to an internal temperature of at least 71°C for not less than 20 min.

Our study also agrees with Bassal et al. (31) and Li et al. (29) who failed to detect HEV RNA in all anti-HEV positive serum samples, which indicates absence of active infection with this virus in their sampled animals. HEV RNA is more likely to be present in young camels’ sera (1–3 years old) than in those of elder animals that have acquired HEV infection at younger age then cleared it by aging (28). Interestingly, Corman et al. (41) described camel meat as less risky in HEV transmission, since the infection in this species occurs in a very young age with a short viremia, while these animals are usually slaughtered at higher ages.

Finally, our study has some limitations that should be taken into consideration. First, the small sample size for a vast zone with big dromedary camels’ population. Second: the absence of recent infection in sampled camels led to the non-detection of any virus RNA in plasma/sera. Thus, further studies must include other samples’ types such as feces and tissues (mainly liver) to increase the sensitivity of HEV RNA detection in camels as well as other animal species that are in close contact with them.

### Conclusion

The current study provides clear evidence of the HEV circulation within the national camelids, as well as the role they may play in the diffusion of this viral infection to humans locally, nationally, and internationally, through direct contact with them and with their derived products or through the food chain.

Additional detailed large-scale spatiotemporal and molecular studies are needed to investigate the prevalence and genotypes of HEV in the national livestock and wildlife, as well as the associated risk factors that interfere with the spread of the infection within animals and humans, in order to implement effective control and prevention approaches in at-risk populations.

## Data availability statement

The original contributions presented in the study are included in the article/supplementary material, further inquiries can be directed to the corresponding author.

## Ethics statement

The animal studies were approved by Scientific and Ethics Committee for Animal Experimentation of the Institute of veterinary sciences, University Frères Mentouri of Constantine 1. The studies were conducted in accordance with the local legislation and institutional requirements. Written informed consent was not obtained from the owners for the participation of their animals in this study because Ethical review and approval was waived for this work, as blood samples were collected before slaughtering. No animals were sacrificed specifically for the purpose of this study.

## Author contributions

AA: Conceptualization, Investigation, Writing – original draft, Writing – review & editing. MB: Resources, Writing – original draft. IB: Resources, Writing – original draft. LD: Investigation, Writing – original draft. SH: Data curation, Writing – original draft. GI: Writing – original draft. MM: Writing – original draft. ID: Conceptualization, Writing – original draft, Writing – review & editing.

## Funding

The author(s) declare financial support was received for the research, authorship, and/or publication of this article. This research was financially co-supported by the Algerian Ministry of Higher Education and Scientific Research (PADESCA Research Laboratory, Institute of Veterinary Sciences, University Frères Mentouri of Constantine 1) and the Italian Istituto Superiore di Sanità (Department of Food Safety, Nutrition and Veterinary Public Health).

## Conflict of interest

The authors declare that the research was conducted in the absence of any commercial or financial relationships that could be construed as a potential conflict of interest.

## Publisher’s note

All claims expressed in this article are solely those of the authors and do not necessarily represent those of their affiliated organizations, or those of the publisher, the editors and the reviewers. Any product that may be evaluated in this article, or claim that may be made by its manufacturer, is not guaranteed or endorsed by the publisher.
